# Risk Factors Associated with Congenital Syphilis, Georgia, 2008-2015

**DOI:** 10.1155/2023/3958406

**Published:** 2023-11-08

**Authors:** Alisa Kachikis, Melissa A. Schiff, Kathryn Moore, Theresa Chapple-McGruder, Jessica Arluck, Jane Hitti

**Affiliations:** ^1^Department of Obstetrics and Gynecology, University of Washington, Seattle, WA, USA; ^2^Department of Internal Medicine, Division of Epidemiology, Biostatistics and Preventive Medicine, University of New Mexico School of Medicine, Albuquerque, NM, USA; ^3^Michigan Medicine, Ann Arbor, MI, USA; ^4^Oak Park Department of Public Health, Oak Park, IL, USA; ^5^Department of Gynecology and Obstetrics, Emory University, Atlanta, GA, USA

## Abstract

**Background:**

Congenital syphilis (CS) is associated with significant perinatal morbidity and mortality. The study objectives were to compare risk factors among women with syphilis infection whose pregnancies did and did not result in CS cases and to evaluate other geographic and socioeconomic characteristics of county of residence as a measure of healthcare inequity.

**Methods:**

This study linked maternal and congenital syphilis data from the Georgia Department of Public Health (DPH), 2008-2015. The Strengthening the Reporting of Observational Studies in Epidemiology (STROBE) reporting guideline was followed. Demographic, behavioral, and case characteristics were compared among women with syphilis infection who did and did not have an infant with CS. Chi-square, Fisher's exact, and multivariate regression analyses were performed using STATA 14.2 (College Station, TX).

**Results:**

Of 505 women with syphilis infection, 23% had an infant with CS, while 77% did not. After adjusting for race/ethnicity, factors associated with CS outcome were age greater than 35 years (adjusted odds ratio (aOR) 3.88; 95% confidence interval (CI) 1.01-14.89), hospital/emergency department diagnosis of syphilis (aOR 3.43; 95% CI 1.54-7.62), and high-risk behaviors such as exchanging sex for money or drugs (aOR 3.25; 95% CI 1.18-8.98). There were no associations between characteristics of county of residence and CS outcome.

**Conclusions:**

This study highlights risk factors that may be associated with CS incidence and the adverse pregnancy outcomes associated with CS. Further work is needed to study improved data collection systems, contributing factors related to CS as well as prevention measures in the United States.

## 1. Introduction

Congenital syphilis (CS), the vertical transmission of *Treponema pallidum* from mother to fetus during pregnancy, is considered a sentinel public health event [1]. Adverse outcomes of untreated syphilis in pregnancy include stillbirth, low birth weight, and congenital infection [2]. Although vertical transmission can be prevented with penicillin treatment at least 30 days prior to delivery, CS remains a global public health problem in the 21^st^ century [3–6]. In the United States (U.S.), rates of CS have fluctuated over the last three decades, steadily increasing in the 2000s, most recently in 2020 to 57.3 cases per 100,000 live births [1, 7], with CS rates mirroring rising trends in primary and secondary syphilis rates among women, especially in the West and South of the U.S. Risk factors cited in published literature for CS include no or late prenatal care (PNC), low socioeconomic status, young age, black race, lack of insurance, single marital status, multiparity, and substance abuse [8–11]. Risk factors for primary and secondary syphilis among heterosexuals, which affect CS rates, also include drug use, high-risk sex practices, and poverty [12–14]. These risk factors for syphilis among reproductive age females and for CS as a group indicate larger health inequities in the US as a common root risk factor.

CS prevention efforts often evaluate cases based on whether they are “missed opportunities” of the healthcare system for screening, diagnosis, treatment, and follow-up of syphilis infection in pregnancy. Approximately 50-60% of all CS cases have had timely entry to PNC and thus could be considered missed opportunities for CS prevention [11, 15–17]. Few studies, however, have attempted to provide a more comprehensive view of potential contributing factors for CS by linking data from maternal records that could possibly indicate sources of healthcare inequities leading to missed opportunities and indirect contribution to these CS cases. Past studies have found examples of possible inequity in access to healthcare, e.g., higher CS rates among rural residents, poorer census tracts, female immigrants, noncitizens, and the uninsured [11, 13, 18–21].

The primary objective of this study was to compare maternal characteristics among women with syphilis infection by the CS status of their infants. We hypothesized that women with syphilis infection and with a pregnancy resulting in a CS case would have increased high-risk behaviors and risk of healthcare inequity as demonstrated by characteristics of the county of residence compared to those without a CS case. We also evaluated pregnancy outcomes among CS cases.

## 2. Materials and Methods

These analyses used surveillance data on maternal and congenital syphilis cases collected by the Georgia Department of Public Health (DPH) from 2008 through 2015. Syphilis and CS are notifiable diseases reported to the Georgia DPH within 24 hours of positive result [22]. The state of Georgia mandates syphilis testing of all pregnant women at initiation of prenatal care and in the third trimester and at the time of delivery of a live-born or a stillborn infant if not previously documented [23]. District and local health departments contact individuals with positive results and investigate cases using the Georgia DPH Syphilis Interview Record form. Interviews were attempted in an estimated 90% of cases of individuals with syphilis in Georgia, and approximately 70% of these were completed [24]. Public health personnel review medical records of all instances of infants born to individuals with syphilis infection and complete the Centers for Disease Control and Prevention (CDC) Congenital Syphilis Case Investigation and Reporting Form according to the CDC surveillance case definition of CS [25]. The CDC surveillance case definition differs from clinical criteria for CS by relying on diagnosis, treatment, and follow-up of the pregnant person and not diagnostic testing. It includes asymptomatic infants and stillbirths, thereby simplifying CS classification and increasing diagnostic sensitivity. Surveillance cases have been shown to have similar risk factors as confirmed cases [25].

For the purposes of this study, maternal surveillance data was linked with infant surveillance data via maternal identification numbers by the Georgia DPH epidemiology department. Surveillance CS cases were not linked with maternal files if testing data was more than one year removed from the time of the CS case (*n* = 1). All linked cases were included regardless of final classification of the CS diagnosis, because each CS surveillance case can potentially identify missed prevention opportunities even if a clinical CS diagnosis in the neonate is not ultimately made [25]. Only deidentified data was available for analysis. The University of Washington Human Subjects Division designated this study as Institutional Review Board (IRB) exempt. This study followed the Strengthening the Reporting of Observational Studies in Epidemiology (STROBE) reporting guideline [26].

To assess our primary objective, we analyzed surveillance data from a cohort of reproductive age individuals who tested positive for syphilis between 2008 and 2015 and who were either pregnant at the time of completion of the Syphilis Interview Form or had been pregnant in the last 12 months. We conducted a nested case control analysis, with cases comprised of individuals with syphilis infection linked to a CS case and controls comprised of individuals with syphilis infection not linked to a CS case. Exposures analyzed were demographic factors associated with disparities in access to healthcare in the U.S., as well as high-risk behaviors recorded on the Syphilis Interview Record form. The demographic risk factors were maternal age, race/ethnicity, and provider type or location of diagnosis. Risk behaviors included exchanging sex for money or drugs in the last 12 months, anonymous sex in the last 12 months, sex while high on drugs in the last 12 months, sex with an injection drug user (IDU) in the last 12 months, and sex with a person known to be a man who has sex with men (MSM) in the last 12 months. A composite of high-risk sexual behaviors in the last 12 months encompassing exchanging sex for money or drugs, anonymous sex, sex while high on drugs, sex with an IDU, and sex with an MSM was also constructed. A composite of high-risk drug use variable was constructed from all drug-related variables (injection drug use in the last 12 months, heroin use in the last 12 months, cocaine use in the last 12 months, crack use in the last 12 months, and methamphetamine use in the last 12 months). Finally, incarceration history in the last 12 months was evaluated.

Additional factors related to poverty and living conditions were examined because of their potential association with healthcare access and inequity. Characteristics examined regarding county of residence included whether or not it was a metropolitan area, and presence or absence of persistent poverty, persistent child poverty, low education, and low employment status as defined by the U.S. Department of Agriculture (USDA) [27]. The USDA uses the U.S. Office of Management and Budget definition of metropolitan areas as central counties with one or more urbanized areas, densely-settled urban areas with populations of 50,000 or more, or outlying counties with 25% or more workers commuting to or from central counties [27, 28]. Persistent poverty and persistent child poverty are defined by the USDA Economic Research Service as counties with 20% or more of the general population or 20% or more of children under 18 years old, persistently poor over three decades as measured by decennial censuses and 5-year estimates for the American Community Survey for 2007-2011 [27, 29, 30]. Counties were designated as low education if 20% or more of its population between 25 to 64 years of age did not have a high school diploma in 2008-2012 and as low employment if less than 65% of 25 to 64-year-old individuals were employed in 2008-2012 [27]. Residence by county based on each of these characteristics (metropolitan, persistent poverty, persistent child poverty, low education, or low employment status) was assessed.

We also summarized demographic characteristics and pregnancy outcomes for infants with CS born 2008-2015 as reported by the Georgia DPH. CS cases not linked with maternal surveillance data were excluded (*n* = 1). Pregnancy outcomes included were gestational age at birth and birth weight.

In our analyses, we compared group characteristics using chi-square and Fisher's exact tests. We performed univariate logistic regression analysis to calculate unadjusted odds ratios (ORs) and 95% confidence intervals (CI) for the demographic and behavioral risk factors of interest. Multivariate logistic regression was used to calculate adjusted ORs and 95% CI for the outcome of CS. The model included all factors that were significantly associated with CS in the univariate analysis: maternal age, provider type or location at diagnosis, and exchange of sex for money or drugs. All cases included in the multivariate analysis did not have missing values for included variables (complete case analysis). The final model also included race/ethnicity since this variable was found to be an important confounder with a change of ≥10% between the crude and adjusted odds ratios. County characteristics, maternal drug use, and incarceration were also evaluated and not found to be important confounders. Cases were excluded from the multivariate analysis if they were missing data on any of the included variables. All analyses were performed using STATA 14.2 (STATACORP, College Station, TX). An alpha level of less than 0.05 was used to determine significance.

## 3. Results

**Figure 1:**
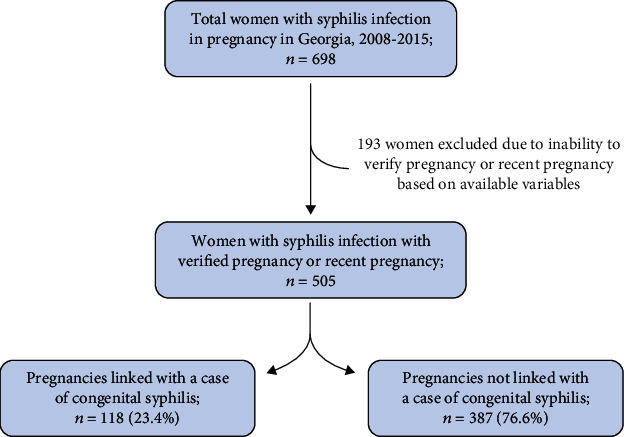
Case selection of pregnant or recently pregnant women with syphilis infection and of congenital syphilis in Georgia, 2008-2015.

Of 698 women with syphilis infection and possible pregnancy reported to the Georgia DPH, 505 women with syphilis infection (72.3%) were confirmed to have a current or recent pregnancy at the time of contact with the Georgia DPH, while 193 cases (27.6%) were excluded due to inability to verify current or recent pregnancy. Of the 505 women with syphilis infection and current or recent pregnancy, 452 (89.5%) were pregnant at the time of interview or exam, 47 (9.3%) were recently pregnant in the last twelve months, and 6 (1.2%) were linked with a case of congenital syphilis within the same year of diagnosis. Of the 505 syphilis-infected women with current or recent pregnancy, 118 (23%) women had an infant with CS, while 387 (77%) women did not (Figure 1). There was one additional infant with CS who could not be linked to a maternal case within 12 months of birth and was therefore excluded.

### 3.1. Demographic and Geographic Characteristics

Among the 505 pregnant women with syphilis infection, 16.2% were younger than 20 years of age, 75.6% were 20-34 years old, and 8.1% were 35 years or older (Table 1). The majority were black (73.9%). Most women lived in metropolitan areas (87.7%), and only a small percentage of women lived in counties with persistent poverty (7.5%), low education levels (8.3%), or low employment levels (16.4%). 49.1% of women lived in counties with persistent child poverty. Women with syphilis infection whose pregnancies did and did not result in a case of CS had similar demographic characteristics and residence by county (Table 1).

### 3.2. Case Characteristics

Location of diagnosis varied between women with and without CS infants (*p* < .001). Those with CS infants were more likely than those with non-CS infants to be diagnosed in hospitals/EDs (47.5 vs 20.4%) and less likely to be diagnosed at a private physician office (28.8% vs 54.5%) or public health clinic (2.7% vs 15.8%).

### 3.3. Behavorial Risk Factors

Women with CS cases were more likely to have exchanged sex for money or drugs and have anonymous sex compared to women without CS cases (6.8% versus 3.1%; *p* = 0.017; and 10.2% versus 6.7%; *p* = 0.044, respectively), although this comparison is limited by missing data. There was no difference between groups in composite high-risk sexual behaviors, composite drug use, or incarceration history.

### 3.4. Multivariate Analysis

In the multivariate analysis, after adjusting for race/ethnicity (Table 2), increased odds of having a CS outcome among pregnant women with syphilis infection was associated with age greater than 35 years (aOR 3.88; 95% CI 1.01-14.89), hospital/ED diagnosis (aOR 3.43; 95% CI 1.54-7.62), and exchanging sex for money or drugs (aOR 3.25; 95% CI 1.18-8.98).

### 3.5. Pregnancy and Neonatal Outcomes

We found that 118 women were linked with cases of infants with CS between 2008 and 2015. Most CS cases were diagnosed at a hospital/ED (*n* = 107, 90.7%). Most were in metropolitan counties (*n* = 103, 87.3%), and a small proportion were in counties with persistent poverty (*n* = 5, 4.2%), low education (*n* = 11, 9.3%), or low employment levels (*n* = 20, 17.0%). The majority of CS cases were diagnosed in counties with persistent child poverty (*n* = 73, 61.9%).

Among 118 women with CS cases, based on maternal and CS case data, 53.4% received PNC with at least one visit, while 19.5% had no PNC. PNC status was not known for 27.1% of cases. Among those with PNC, 17.5% had 1-3 visits, 30.2% had 4-10 visits, and 12.7% had greater than 10 visits, while the number of visits was unknown for 39.7% of women. Regarding treatment timing, among 118 cases, 2 women (1.7%) were treated prior to pregnancy, 28 (23.7%) were treated during pregnancy, 52 (44.1%) had no treatment, and 36 (30.5%) had missing responses. Of the 2 women treated before pregnancy, both infants had positive nontreponemal and treponemal test results. Among women treated during pregnancy, 64.3% received treatment less than 30 days prior to delivery. Among 118 CS cases, 9 (7.6%) were stillbirths, and 109 (92.4%) were live births. Among live-born infants, 99.1% and 85.3% received nontreponemal and treponemal testing, respectively. Darkfield microscopy or direct fluorescent antibody testing were not performed in any of the cases. IgM–specific treponemal tests, long bone X-rays, and CSF evaluations were performed in 7.3%, 40.4%, and 61.5% of cases, respectively (above data not shown in tables). In terms of overall pregnancy outcomes, among live-born infants, 38 (34.9%) babies were born prematurely before 37 weeks gestational age, and 36 (33.0%) of live-born babies had birth weights less than 2500 grams.

## 4. Discussion

This study examines risk factors for CS births among women with syphilis in pregnancy and evaluates the effect of PNC on pregnancy outcomes among infants with CS. One of the major findings of this analysis was a significant amount of missing responses which limit the interpretation of findings (see Table 1). While our study results must be evaluated cautiously given the amount of missing data, certain findings were consistent with prior studies. We found increased odds of having a CS infant in women who engaged in high-risk behaviors such as exchanging sex for money or drugs. A study in Arizona found that exchanging sex for money or drugs was a significant risk factor for syphilis infection in pregnant women [11]. These high-risk behaviors may signal or be associated with other social factors or “social vulnerabilities” that adversely impact the ability of a woman to access care and may necessitate additional social support [31]. Sex workers may experience gender-based violence that adversely affects pregnancy outcomes and may decrease access to care. Sex workers may also encounter institutional barriers even in settings with universal health coverage [32, 33]. Among pregnant women with syphilis, older women had higher odds of having a CS pregnancy outcome. This may be related to increased parity among women of older age; however, information related to parity was not available in our database. One study from Georgia found that older maternal age was associated with CS, while another study in New York found an association with increased parity, but not increased age [15, 34]. Few studies compare pregnant women with syphilis and with and without CS outcome; however, the similar characteristics between the two groups may indicate that women who test positive for syphilis during pregnancy regardless of CS outcome are more similar to each other than they are to pregnant women who test negative for syphilis [35].

Our finding that most women with CS cases were diagnosed in hospitals/EDs may represent delay or barriers in access to care; however, the majority of women with CS outcome did receive some PNC. Similar findings have been shown in other studies on missed opportunities in preventing CS [15, 16]. Despite the large amount of missing data, our study suggests that women with syphilis and CS cases are often diagnosed in hospitals and EDs and may have had contact with DPH later in pregnancy, emphasizing the importance of the DPH in facilitating access to testing and treatment and working with health facilities to provide syphilis care and follow-up as well as education to healthcare providers and patients [36].

Our summarized findings of pregnancy outcomes among the cases of CS including preterm delivery and low birth weight are consistent with known adverse outcomes of pregnancies complicated by syphilis infection [2]. These outcomes are likely multifactorial, and we were unable to assess clinical comorbidities and socioeconomic factors not captured by the CS intake form.

Given limitations in the available data for this analysis, we were unable to directly assess healthcare equity, or associations between equity and syphilis in pregnancy or CS outcome. Overall, demographic characteristics did not differ significantly between groups in our analyses. When evaluating county characteristics, most women lived in metropolitan counties without persistent poverty, low education, or low employment. This was also the case in the analysis of CS cases. Of note, around 50% of syphilis-positive pregnant women lived in counties with persistent child poverty, and over 50% of CS cases were diagnosed in these counties, indicating a potential association between socioeconomic factors and CS. In order to fully evaluate healthcare equity and barriers to care, it is important to evaluate what data is being and should be collected. The DPH's surveillance data on syphilis and CS cases focuses on behavioral risk factors and diagnostic criteria that are influenced by state public health systems. By its nature, this data provides little or no information about social and gender-based determinants of health. Furthermore, CS cases should all be considered “missed opportunities” in accessibility of the healthcare system to high-risk women and in the testing, diagnosis, and treatment of syphilis in pregnancy.

This study is limited by missing data, which may have affected the analyses and decreased the ability to find differences in demographic and risk factors between groups. Differentials in missing data between groups can also introduce bias in regards to the effect size of risk factors or direction of outcomes [37]. Another limitation is the small sample size, limiting the power to detect differences in groups and to adjust for potential confounders. In addition, pregnancy outcomes of pregnant women with syphilis not linked to a CS case were not known. This limits the comparison of women with and without a CS case; some women may have experienced syphilis-associated early pregnancy losses. Another limitation in the analysis of CS cases is the lack of clinical information such as maternal obstetric history and comorbid conditions which may impact pregnancy outcomes. Finally, reasons for lack of PNC among the CS cases are unknown, limiting understanding of important differences between cases with and without PNC [38].

## 5. Conclusion

This study highlights several risk factors that may be associated with CS incidence and adverse pregnancy outcomes in the state of Georgia. It also emphasizes the importance of state DPH work and prompt reporting of positive test results to the DPH by testing centers, as well as the need for close collaboration between health providers and the DPH in the response to cases of syphilis in pregnancy. In addition, education of providers at access points and of women in communities on the importance of PNC is paramount to achieving healthy pregnancy outcomes. Future studies are needed to improve the data available related to CS cases and to assess additional factors related to healthcare inequity that may impact CS and allow the identification of opportunities for CS prevention.

## Figures and Tables

**Table 1:** Comparison of demographic and behavioral risk factors and case characteristics among pregnant women with syphilis infection that did and did not result in a case of congenital syphilis (CS) in Georgia, 2008-2015.

	Total	Pregnancy outcome with CS	Pregnancy outcome without CS	*p* value
	*n* = 505	*n* = 118	*n* = 387	
*Demographic characteristics*	*n* (%)	*n* (%)	*n* (%)	
Maternal age				0.07
<20 years	82 (16.2)	12 (10.2)	70 (18.1)	
20-34 years	382 (75.6)	93 (78.8)	289 (74.7)	
35+ years	41 (8.1)	13 (11.0)	28 (7.2)	
*Missing response*	*0*	*0*	*0*	
Maternal race/ethnicity^‡^				0.93
White, non-Hispanic	71 (14.1)	18 (15.2)	53 (13.7)	
Black, non-Hispanic	373 (73.9)	87 (73.7)	286 (73.9)	
Asian/Other	8 (1.6)	1 (0.8)	7 (1.8)	
Hispanic	52 (10.3)	12 (10.2)	40 (10.3)	
*Missing response*	*1 (0.2)*	*0*	*1 (0.3)*	
*Geographic characteristics^§^ and provider type/location at diagnosis^§^*				
County—Metropolitan				0.64
No	58 (11.5)	12 (10.2)	46 (11.9)	
Yes	443 (87.7)	104 (88.1)	339 (87.6)	
*Missing response*	*4 (0.8)*	*2 (1.7)*	*2 (0.5)*	
County—persistent poverty^‡^				0.07
No	463 (91.7)	112 (94.9)	351 (90.7)	
Yes	38 (7.5)	4 (3.4)	34 (8.8)	
*Missing response*	*4 (0.8)*	*2 (1.7)*	*2 (0.5)*	
County—persistent child poverty^‡^				0.93
No	253 (50.1)	59 (50.0)	194 (50.1)	
Yes	248 (49.1)	57 (48.3)	191 (49.4)	
*Missing response*	*4 (0.8)*	*2 (1.7)*	*2 (0.5)*	
County—low education level				0.38
No	459 (90.9)	104 (88.1)	355 (91.7)	
Yes	42 (8.3)	12 (10.2)	30 (7.8)	
*Missing response*	*4 (0.8)*	*2 (1.7)*	*2 (0.5)*	
County—low employment level				0.36
No	418 (82.8)	100 (84.8)	318 (82.2)	
Yes	83 (16.4)	16 (13.6)	67 (17.3)	
*Missing response*	*4 (0.8)*	*2 (1.7)*	*2 (0.5)*	
Provider type/location at syphilis diagnosis				<0.001
Public health clinic	76 (15.0)	15 (2.7)	61 (15.8)	
Private physician	245 (48.5)	34 (28.8)	211 (54.5)	
Hospital/ED	135 (26.7)	56 (47.5)	79 (20.4)	
Other	29 (5.7)	7 (5.9)	22 (5.7)	
*Missing response*	*20 (4.0)*	*6 (5.1)*	*14 (3.6)*	
*Behavioral risk factors*				
In last 12 months, exchanged sex for money or drugs				0.02
No	408 (80.8)	75 (63.6)	333 (86.0)	
Yes	20 (4.0)	8 (6.8)	12 (3.1)	
*Missing response*	*77 (15.2)*	*35 (29.7)*	*42 (10.8)*	
In last 12 months, had anonymous sex				0.04
No	393 (77.8)	71 (60.2)	322 (83.2)	
Yes	38 (7.5)	12 (10.2)	26 (6.7)	
*Missing response*	*74 (14.6)*	*35 (29.7)*	*39 (10.1)*	
In last 12 months, had sex while high on drugs				0.87
No	355 (70.3)	68 (57.6)	287 (74.2)	
Yes	70 (13.9)	14 (11.9)	56 (14.5)	
*Missing response*	*80 (15.8)*	*36 (30.5)*	*44 (11.4)*	
In last 12 months, had sex with an IV drug user^‡^				0.59
No	424 (84.0)	83 (70.3)	341 (88.1)	
Yes	5 (1.0)	0 (0)	5 (1.3)	
*Missing response*	*76 (15.0)*	*35 (29.7)*	*41 (10.6)*	
In last 12 months, had sex with an MSM^‡^				0.58
No	418 (82.8)	80 (67.8)	338 (87.3)	
Yes	4 (0.8)	1 (0.8)	3 (0.8)	
*Missing response*	*83 (16.4)*	*37 (31.4)*	*46 (11.9)*	
High-risk sex behavior in last 12 months (composite)^¶^				0.43
No	328 (65.0)	60 (50.8)	268 (69.2)	
Yes	91 (18.0)	20 (17.0)	71 (18.4)	
*Missing response*	*86 (17.0)*	*38 (32.2)*	*48 (12.4)*	
Incarcerated in the last 12 months				0.21
No	389 (77.0)	74 (62.7)	315 (81.4)	
Yes	36 (7.1)	10 (8.5)	26 (6.7)	
*Missing response*	*80 (15.8)*	*34 (28.8)*	*46 (11.9)*	
IV drug use in the last 12 months^‡^				0.26
No	422 (83.6)	82 (69.5)	340 (87.9)	
Yes	5 (1.0)	2 (1.7)	3 (0.8)	
*Missing response*	*78 (15.4)*	*34 (28.8)*	*44 (11.4)*	

Abbreviations: ED: emergency department; IV: intravenous; MSM: men who have sex with men. ^†^For the variable “Gestational age at interview/exam,” the number of women who were pregnant at the interview or exam was 424. ^‡^Comparison performed with the Fisher exact test. ^§^Data related to county and provider type/location of diagnosis analyzed in this table was collected using the Syphilis Interview Record form and may not be identical to the congenital syphilis surveillance data recorded using the CDC Congenital Syphilis (CS) Case Investigation and Reporting Form. ^¶^Included in the composite variable for “High risk sex behaviour in last 12 months” were the following variables: “In last 12 months, exchanged sex for money or drugs,” “In last 12 months, had anonymous sex,” “In last 12 months, had sex while high on drugs,” “In last 12 months, had sex with an IV drug user,” and “In last 12 months, had sex with an MSM.” “Yes” denotes a yes response to any of the variables. “No” denotes a no response to all of the variables.

**Table 2:** Multivariable analysis of demographic and behavioral risk factors and case characteristics of pregnant women with syphilis infection with a comparison of pregnancies that did and did not result in a case of congenital syphilis (CS) in Georgia, 2008-2015.

	CS case^†^ aOR^‡^	95% CI
Maternal age		
<20 years	1.00	Referent
20-34 years	4.22	1.57-11.37
35+ years	3.88	1.01-14.89
Provider type/location at diagnosis		
Public health clinic	1.00	Referent
Private physician	0.67	0.30-1.51
Hospital/ED	3.43	1.54-7.62
Other	1.25	0.39-4.01
In last 12 months, exchanged sex for money or drugs		
No	1.00	Referent
Yes	3.25	1.18-8.98

Abbreviations: aOR: adjusted odds ratio; CI: confidence interval; CS: congenital syphilis; ED: emergency department. ^†^The reference group for this analysis was pregnant women with syphilis infection without a CS case. ^‡^The OR was adjusted for all other variables in the table as well as race/ethnicity.

## Data Availability

Data on syphilis cases in Georgia may be requested through the Georgia Department of Public Health.
